# Peptide-Based Inorganic Nanoparticles as Efficient Intracellular Delivery Systems

**DOI:** 10.3390/pharmaceutics17091123

**Published:** 2025-08-28

**Authors:** Amir Nasrolahi Shirazi, Rajesh Vadlapatla, Ajoy Koomer, Anthony Nguyen, Vian Khoury, Keykavous Parang

**Affiliations:** 1 Department of Pharmaceutical Sciences, College of Pharmacy, Marshall B. Ketchum University, 2575 Yorba Linda Blvd., Fullerton, CA 92831, USA; rvadlapatla@ketchum.edu (R.V.); akoomer@ketchum.edu (A.K.); anthonynguyen.cop28@ketchum.edu (A.N.); viankhoury.cop26@ketchum.edu (V.K.); 2 Center for Targeted Drug Delivery, Department of Biomedical and Pharmaceutical Sciences, Chapman University School of Pharmacy, Harry and Diane Rinker Health Science Campus, 9401 Jeronimo Rd, Irvine, CA 92618, USA; parang@chapman.edu

**Keywords:** drug delivery systems, gadolinium nanoparticles, gold nanoparticles, inorganic nanoparticles, peptide nanoparticles, selenium nanoparticles

## Abstract

**Background/Objectives**: Peptide-based inorganic nanoparticles (PINPs) have emerged as promising candidates for intracellular delivery due to their unique structural and functional attributes. These hybrid nanostructures combine the high surface area and tunable optical/magnetic properties of metal cores (e.g., Au, Ag, Fe_3_O_4_) with the biocompatibility, targeting specificity, and responsive behavior of peptides. In particular, peptides with amphipathic or cell-penetrating features could facilitate efficient transport of molecular cargos across cellular membranes while enabling stimulus-responsive drug release in target tissues. **Methods**: We review key synthesis methods (especially green, peptide-mediated one-pot approaches), functionalization strategies (e.g., thiol-gold bonds, click chemistries), and characterization techniques (TEM, DLS, FTIR, etc.) that underpin PINP design. In addition, we highlight diverse peptide classes (linear, cyclic, amphipathic, self-assembling) and their roles (targeting ligands, capping/stabilizing agents, reducing agents) in constructing multifunctional nanocarriers. **Results**: The prospects of PINPs are considerable: they enable targeted drug delivery with imaging/theranostic capability, improve drug stability and cellular uptake, and harness peptide programmability for precision nanomedicine. However, challenges such as in vivo stability, immunogenicity, and standardization of evaluation must be addressed. **Conclusions**: Overall, PINPs represent multifunctional platforms that could significantly advance drug delivery and diagnostic applications in the future.

## 1. Introduction

Nanotechnology is a multidisciplinary field that involves the design, synthesis, and manipulation of materials at the nanometer scale, typically between 1 and 100 nm, to exploit unique physicochemical properties not observed in their larger counterparts. At this scale, materials often exhibit enhanced surface area, quantum effects, and novel mechanical, optical, or catalytic behaviors, making them highly suitable for biomedical, environmental, and technological applications [1,2].

Targeting specificity remains a major challenge. Many chemotherapeutic agents, for example, are toxic to both cancerous and healthy cells, leading to severe side effects. Nanotechnology offers transformative potential across various aspects of drug delivery. Achieving targeted delivery, whether passive by nanoparticles (e.g., enhanced permeability and retention (EPR) effect in tumors) or active (e.g., ligand–receptor mediated), is essential to improving therapeutic outcomes and reducing adverse effects [3].

The EPR effect serves as a foundational mechanism for the passive accumulation of nanoparticles in solid tumors. Tumor vasculature typically exhibits fenestrations ranging from 100 to 800 nm, allowing nanoparticles between 10 and 100 nm to infiltrate tumor tissue while minimizing accumulation in healthy tissue [4].

Among nanomaterials, metal nanoparticles (MNPs) have attracted significant attention due to their distinctive size-dependent properties and versatile functionality. MNPs offer distinct advantages in drug delivery, including tunable size, high surface area, multifunctionality, and the ability to be engineered for controlled release, targeting, and imaging. These nanoparticles, composed of metals such as gold (Au), silver (Ag), iron oxide (Fe_3_O_4_), platinum (Pt), and zinc oxide (ZnO), can be synthesized through a variety of chemical, physical, or biological approaches. Among the most widely studied MNPs are gold (AuNPs), silver (AgNPs), iron oxide (Fe_3_O_4_ NPs), zinc oxide (ZnO NPs), selenium (SeNPs), and gadolinium (GdNPs), each with unique properties suitable for different therapeutic and diagnostic applications. AuNPs and AgNPs in particular, have been extensively studied for their optical properties, including surface plasmon resonance (SPR), which enables applications in bioimaging, diagnostics, and photothermal therapy [5,6].

From targeting delivery to cancer therapy, by leveraging the unique physiochemical properties of MNPs, scientists can develop effective and targeted therapeutic strategies. However, it comes at a cost of resolving challenges such as potential toxicity, limited production, and higher instability. Ongoing interdisciplinary investigations will be essential to fully understand key requirements for precise targeting, enhanced pharmacokinetics, and biocompatibility of metal nanoparticles in biomedical applications.

AuNPs are favored for their stability, biocompatibility, and surface plasmon resonance properties. They are easily functionalized with drugs, peptides, or targeting ligands for tumor-selective delivery and are widely used in photothermal therapy and imaging [6]. Functionalized AuNPs improve drug solubility, tumor accumulation, and reduce systemic side effects [7].

In addition, AgNPs are well-known for their potent antimicrobial activity and are helpful in wound healing and infectious disease applications. They also have anti-inflammatory and anticancer effects due to their ability to generate reactive oxygen species (ROS) and disrupt cellular membranes [8]. AgNPs are co-loaded with antibiotics or anticancer agents in drug delivery for synergistic effects.

Fe_3_O_4_ NPs possess superparamagnetic properties, allowing for magnetic targeting and MRI contrast enhancement. Coated with polymers or peptides, they can deliver drugs to tumors or inflamed tissues under external magnetic fields [9]. Their biocompatibility and biodegradability make them ideal for multifunctional theranostic applications. In addition, Fe_3_O_4_ NPs have been shown to not only enable a favorable release profile for the anticancer drug 5-fluorouracil (5-FU) but also to overcome barriers related to cellular membrane penetration [10].

ZnO NPs are pH-sensitive carriers that disintegrate in acidic tumor environments, promoting localized drug release and ROS generation [11]. Their selective cytotoxicity and potential to induce apoptosis make them promising candidates for cancer therapy.

SeNPs have recently gained attention due to their antioxidant, anticancer, and immunomodulatory effects. SeNPs can inhibit tumor cell proliferation by inducing apoptosis and modulating redox homeostasis while exhibiting minimal toxicity to normal cells [12]. Their surface can be functionalized with targeting ligands or drugs for enhanced specificity and efficacy. SeNPs have shown promise in delivering chemotherapeutics and natural compounds for cancer, cardiovascular, and inflammatory diseases.

GdNPs are primarily known for their role in magnetic resonance imaging (MRI) due to the paramagnetic properties of gadolinium ions. However, GdNPs are increasingly being engineered as theranostic agents, combining diagnostic imaging with drug delivery or photothermal therapy [13]. Their ability to serve as contrast agents while delivering therapeutic payloads enhances precision and real-time monitoring, especially in oncology.

Thus, MNPs provide diverse platforms for drug delivery and diagnosis. Their size, surface properties, and intrinsic functionalities can be finely tuned, making them valuable in the development of safe, effective, and personalized nanomedicine strategies. Table 1 summarizes common MNP types with their unique properties and biomedical uses, as noted above.

## 2. Key Physicochemical and Biological Properties of MNPs

### 2.1. Size of MNPs

The size of MNPs is a critical determinant in their biological performance and therapeutic potential in drug delivery systems. Nanoparticles typically range between 1 and 100 nm, and slight differences in this range can lead to significant variations in biodistribution, circulation time, cellular uptake, clearance, and toxicity [1,2]. Optimizing nanoparticle size is essential for enhancing drug delivery efficacy and minimizing side effects.

### 2.2. Surface Functionalization

**Figure 1:**
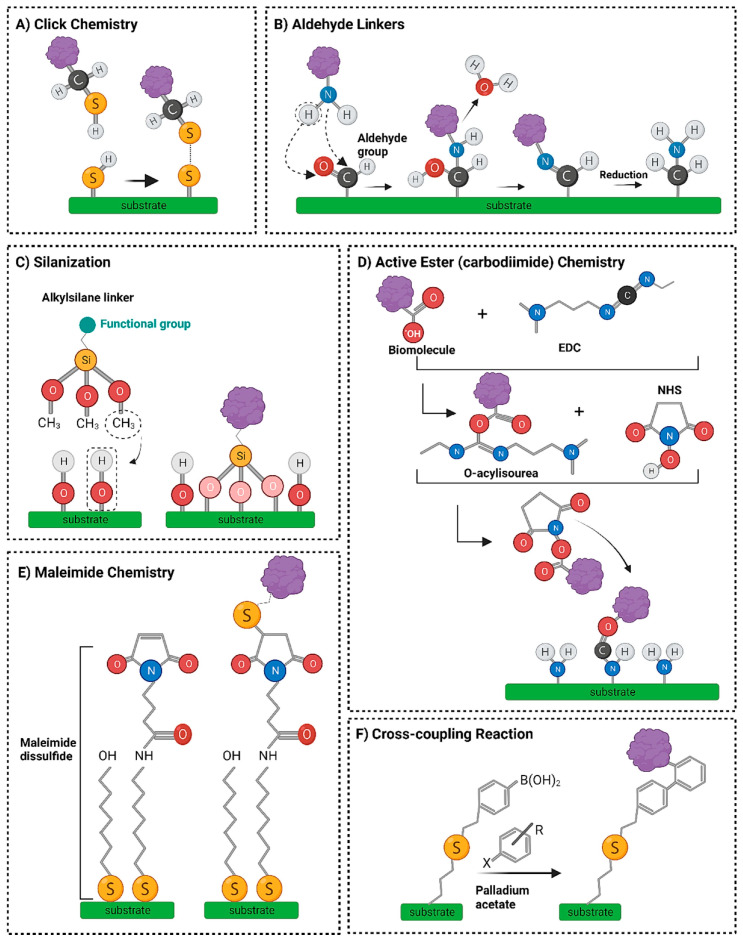
Schematic illustration of chemical reactions explored in this article: some popular surface bio-modification strategies. (**A**) Click reaction: Thiol functional groups engage in transient S-S bond formation, which can subsequently undergo reduction in an acidic environment. (**B**) The covalent binding of proteins to substrate aldehyde groups leads to the formation of transient amine linkages. A reduction reaction facilitates the creation of a covalent N-C bond. (**C**) Silanization: Silane linkers undergo condensation reactions with hydroxylated substrates, resulting in the formation of H_3_COH. (**D**) Active ester carbodiimide reactions: 1-Ethyl-3-(3-dimethylaminopropyl)carbodiimide (EDC) forms a C-O bond by interacting with carboxyl functional groups of biomolecules. To enhance conjugation efficiency, *N*-hydroxysuccinimide (NHS) is subsequently introduced. The amine functional groups of the substrate then replace NHS in the reaction. (**E**) Synthesis of self-assembled monolayer (SAMs) and bioconjugation using maleimide-thiol chemistry: Maleimide-terminated alkane disulfides in pure solutions are employed to prepare maleimide-terminated SAMs, facilitating bioconjugation with biomolecules containing thiol groups. (**F**) Functionalization of mesoporous silica gel using various organic functional groups via Suzuki coupling with aromatic halides and thiol-ene coupling of surface thiol groups with 4-vinylphenylboronic acid. Created with BioRender.com. Reprinted with permission [22]. Copyright 2023 Science Direct, Elsevier.

One of the most compelling advantages of MNPs is their surface modifiability. Their surfaces can be functionalized with various biomolecules such as antibodies, peptides, or polymers to improve biocompatibility, target specificity, and circulation time in the body [20]. Additionally, the high surface-area-to-volume ratio of MNPs enhances their drug-loading capacity, making them excellent platforms for targeted drug delivery systems [21]. Figure 1 shows various chemical reactions that are used to functionalize nanoparticles using Click chemistry, aldehyde linkers, silanization, carbodiimide chemistry, maleimide chemistry, or cross-coupling [22].

### 2.3. Surface Charge

Surface charge is one of the most impactful features that can determine the properties of nanoparticles. Due to the high complexity of human physiological fluids, most studies investigating the physicochemical properties of nanoparticles in such environments are conducted using in vitro models. Surface charge helps prevent nanoparticle aggregation by generating repulsive forces that counteract natural attractions like van der Waals forces. This charge is not intrinsic but depends on the surrounding medium.

The surface charge of naked nanoparticles could change upon functionalization processes with ligands such as amino or hydroxyl groups to become positive or negative. In addition, surface charge could be a function of the pH value and change in different environments [23].

### 2.4. Toxicity Considerations

Despite their promise, MNPs also pose challenges, particularly related to toxicity, stability, and environmental concerns. The interaction of these nanoparticles with biological systems depends on multiple factors such as size, shape, surface charge, and coating materials. For instance, unmodified or poorly stabilized MNPs may induce oxidative stress or inflammatory responses, limiting their clinical translation [24]. Hence, designing MNPs with tunable, stable, and biocompatible properties remains a crucial area of ongoing research in nanomedicine. The integration of MNPs with peptides, known for specificity, biodegradability, and low immunogenicity, could enhance their potential, particularly in drug delivery and precision medicine.

## 3. Significance of Combining Peptides with MNPs for Different Applications

**Figure 2:**
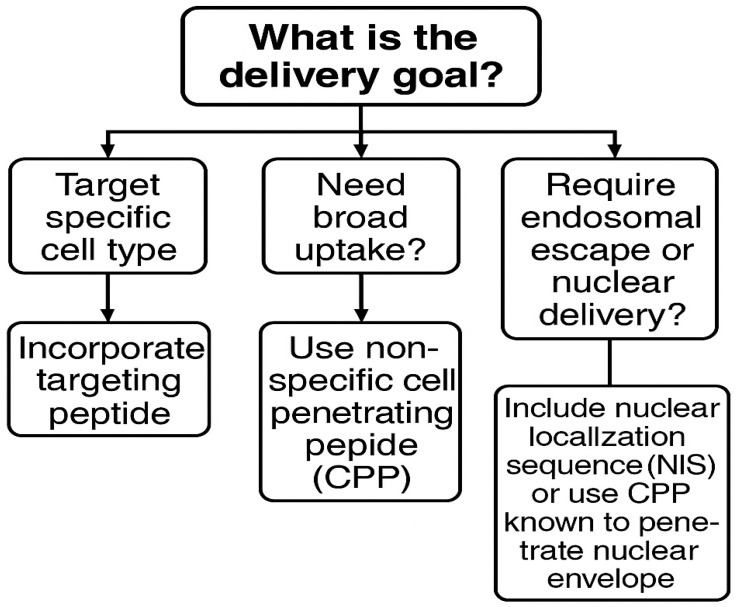
Decision tree for selecting peptide classes based on delivery goals.

The combination of peptides with MNPs has emerged as a highly promising strategy for a wide range of biomedical and technological applications due to the synergistic advantages conferred by both components [25,26,27,28]. Peptides, known for their biological compatibility, structural diversity, and target specificity, can be engineered to interact selectively with cells, receptors, or environmental stimuli [29,30]. Peptides possess distinct intrinsic properties and can be strategically selected according to the specific delivery goal, as illustrated in the decision tree in Figure 2. These concepts are explained in the next sections.

One of the most critical applications of peptide-based inorganic nanoparticles (PINPs) is in targeted drug delivery. The peptides serve as homing ligands that recognize and bind to specific receptors overexpressed on diseased cells, enabling precise delivery of therapeutics while minimizing systemic toxicity [31]. Additionally, these constructs offer improved penetration and cellular uptake due to the amphipathic or cell-penetrating nature of many peptides [32]. For example, the integration of tumor-homing peptides with AuNPs has led to significant improvements in the selective accumulation of anticancer agents at tumor sites, enhancing therapeutic outcomes [33].

In diagnostics and biosensing, PINPs have proven valuable for their specificity and sensitivity. MNPs exhibit unique optical and electronic properties, including SPR, which can be modulated upon peptide–target interactions, allowing for real-time and label-free detection of biomolecules [34]. Moreover, antimicrobial applications of peptide-coated AgNPs leverage the inherent antibacterial activity of silver with the selective targeting and membrane-disrupting effects of antimicrobial peptides (AMPs), thus reducing the required dosage and minimizing resistance development [35].

The use of peptides also enhances the colloidal stability and biocompatibility of MNPs. Peptides can act as capping or templating agents during nanoparticle synthesis, controlling size and morphology while preventing aggregation in physiological environments [34]. Furthermore, in theranostic systems, where diagnosis and therapy are combined, peptides facilitate multifunctionality by linking therapeutic, imaging, and targeting modalities into a single nanostructure [36].

Thus, integration of peptides with MNPs harnesses the molecular recognition capacity of peptides with the functional attributes of MNPs, resulting in highly customizable platforms for use in drug delivery, biosensing, imaging, and antimicrobial strategies. This combinatorial approach continues to drive innovation in nanomedicine and other interdisciplinary fields.

This review aims to provide a comprehensive overview of the integration of peptides with MNPs, emphasizing their synergistic roles across various biomedical and technological domains. The framework includes the fundamental principles of peptide–MNP conjugation, including synthesis strategies, physicochemical properties, and structural interactions. Special attention is given to their applications in drug delivery, diagnostics, antimicrobial therapy, and biosensing, where the unique attributes of peptides, such as biocompatibility, target specificity, and structural versatility, enhance the functionality and efficacy of metal nanomaterials. Furthermore, this review explores current challenges, such as toxicity, stability, and translational barriers, and it discusses emerging trends and future perspectives. The objective is to highlight the interdisciplinary potential of peptide-MNP hybrids and to guide future research toward more effective and targeted nanomedical solutions.

## 4. Peptides in Peptide-Based Inorganic Nanoparticles (PINP) Technology

### 4.1. Types of PINPs Used According to the Peptide Structure

Peptides are increasingly utilized in nanotechnology due to their tunable structure, biocompatibility, and ability to confer specificity and functionality to nanomaterials [29,37]. Based on their structure, peptides can be broadly classified into linear, cyclic, amphipathic, and self-assembling peptides. Each type offers distinct advantages for nanotechnological applications, particularly when conjugated with MNPs.

Researchers can design highly specialized nanoplatforms by carefully choosing peptide structures based on their conformational and functional characteristics. This structural versatility is critical in determining nanoparticle stability, targeting efficiency, and biological interactions, thereby expanding the applications of PINPs in medicine, diagnostics, and material science. Table 2 below summarizes these structural peptide classes and their roles in PINP systems.

#### 4.1.1. Linear Peptide Use in PINPs

Linear peptides, composed of amino acids linked in a sequential, unbranched chain, play a pivotal role in nanotechnology due to their ease of synthesis, modifiability, and functional versatility. Their straightforward structure allows precise control over sequence composition and length, making them ideal candidates for surface modification, targeting, drug delivery, and nanoparticle stabilization. The integration of linear peptides with nanomaterials, particularly MNPs, has significantly enhanced these nanostructures’ specificity, bioactivity, and functionality in various biomedical applications.

Yin et al. developed a streamlined method for preparing AuNPs that are functionalized with RGD peptides capable of targeting tumor cells. The innovation lies in using either linear RGD or cyclic RGD (cRGDyE) peptides to simultaneously reduce, stabilize, and target the AuNPs in a single reaction, eschewing the need for multiple reagents and steps [38].

These RGD–AuNPs were shown to be colloidally stable, biocompatible, and effective targeting agents. Microscopy (dark-field light scattering, transmission electron microscopy (TEM)) demonstrated selective uptake by A375 melanoma cells, which overexpress integrin α_vβ_3, as opposed to MCF-7 breast cancer cells with low integrin levels. A competitive inhibition assay further confirmed that cellular uptake was integrin mediated. Quantitative comparisons suggested the cyclic RGD–AuNPs exhibited slightly stronger targeting efficiency than their linear counterparts, although both retained full peptide activity post-conjugation.

In nanoparticle synthesis and stabilization, linear peptides can serve as capping or templating agents. For instance, peptides with specific amino acid residues (e.g., cysteine, histidine, lysine) can coordinate with metal ions and assist in the nucleation and controlled growth of nanoparticles such as gold or silver [39]. These peptides regulate particle size and shape, providing colloidal stability under physiological conditions.

#### 4.1.2. Cyclic Peptide Use in PINPs

Cyclic peptides have attracted growing interest in nanotechnology due to their enhanced stability, receptor-binding affinity, and structural rigidity compared to their linear counterparts [26,27]. These peptides are characterized by a covalently closed-loop structure, formed either through head-to-tail cyclization or via side-chain linkages such as disulfide bonds or lactam bridges. This cyclization imparts resistance to proteolytic degradation and conformational flexibility, making cyclic peptides highly suitable for biomedical nanotechnology applications, especially in targeted drug delivery, molecular recognition, and nanoparticle functionalization.

Shirazi et al. developed a novel and green method for synthesizing AuNPs using cyclic peptides composed of alternating arginine and tryptophan residues, specifically [WR]_3–5_. These peptides served dual functions: tryptophan residues acted as mild reducing agents, while arginine residues stabilized the nanoparticles via electrostatic interactions. Among the variants, [WR]_4_-AuNPs demonstrated optimal size and stability and were evaluated for their drug delivery potential. The study showed that these peptide-capped AuNPs significantly enhanced cellular uptake, up to 15-fold, of antiviral drugs such as lamivudine and emtricitabine in SK-OV-3 and CCRF-CEM cancer cell lines [25,26]. Similar outcomes were observed by nanoparticles (SeNPs) [27] and gadolinium nanoparticles (GdNPs) [28].

**Figure 3:**
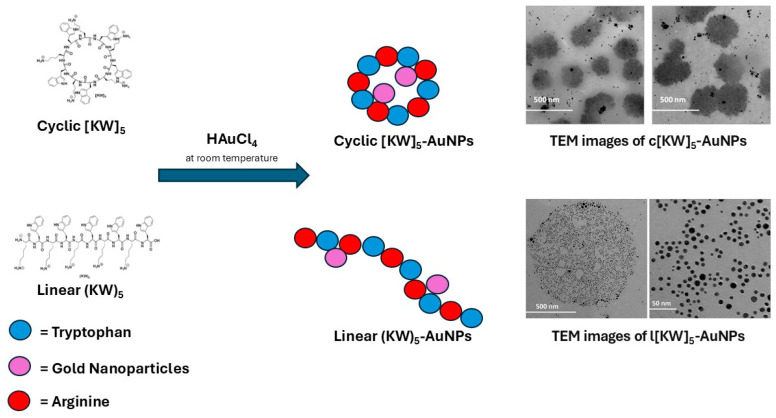
Linear versus cyclic peptide gold nanoparticles. Reprinted with permission [25]. Copyright 2013 American Chemical Society. Cyclic peptide containing arginine and tryptophan-based metal nanoparticles.

This class of peptides showed that cyclic peptides behave differently in producing and stabilizing nanoparticles, and their ability to cross the cell membrane was found to be different. TEM data showed that the morphology of linear peptide containing lysine and arginine-AuNPs is visually distinct from that of their cyclic peptide-AuNP counterpart (Figure 3).

In addition, their potency to deliver drugs across the cell membrane was found to be more prominent with the cyclic counterparts [24]. Cyclic peptides containing arginine and tryptophan were investigated with gadolinium and selenium nanoparticles. Cellular uptake studies showed that cyclic peptide–MNPs were even more effective tools for delivering drugs intracellularly than their linear counterparts and peptides alone [27,28].

#### 4.1.3. Amphipathic Peptide Use in PINPs

Amphipathic peptides, characterized by their spatial segregation of hydrophilic and hydrophobic residues, play a critical role in nanotechnology due to their ability to interact with both aqueous environments and lipid or polymeric systems. Their unique structural features allow them to interface effectively with cell membranes, self-assemble into nanostructures, and stabilize nanocarriers. As a result, amphipathic peptides have found wide-ranging applications in drug delivery, membrane penetration, nanoparticle functionalization, and the design of responsive nanomaterials [29].

A major application of amphipathic peptides is cell-penetrating peptides (CPPs), which are widely used to facilitate intracellular delivery of nanoparticles and therapeutic cargos. Classic examples include the TAT peptide from HIV-1 and penetratin derived from the Antennapedia homeodomaintranslocate across cellular membranes [29,30]. When conjugated to MNPs, amphipathic peptides significantly improve cellular uptake and intracellular trafficking, particularly for hard-to-deliver cargos such as siRNA, DNA, and proteins [31,32,33].

#### 4.1.4. Self-Assembling Peptide Use in PINPs

Self-assembling peptides represent a unique class of biomolecules that can spontaneously organize into highly ordered nanostructures such as nanofibers, nanotubes, nanospheres, and hydrogels through non-covalent interactions, including hydrogen bonding, hydrophobic interactions, and π–π stacking. Their intrinsic ability to form predictable and tunable nanostructures makes them highly attractive for various nanotechnology applications, including drug delivery, tissue engineering, biosensing, and nanofabrication [30].

Self-assembling peptides form scaffolds (nanofibers, hydrogels) that nucleate metal deposition. For instance, engineered peptide fibrils with surface cysteines have been used as templates to grow metal nanowires. Peptide hydrogels have also been shown to generate AgNPs in situ under light, forming hybrid biomaterials with enhanced stability and antibacterial function [34]. The information is summarized in Table 2. In addition, Table 3 presents a comparative analysis of peptide morphologies (linear, cyclic, and amphipathic) and their influence on PINP behavior across key delivery stages, including membrane binding, endocytosis, and cytosolic release.

In summary, the classification of peptides into categories such as cell-penetrating, targeting, amphipathic, cyclic, or stimuli-responsive is not merely a taxonomic exercise based on sequence or structure; it also reflects fundamental differences in how these molecules interact with cellular membranes, receptors, and intracellular compartments, thereby dictating their predominant delivery pathways.

Cationic cell-penetrating peptides, enriched in positively charged amino acids such as arginine or lysine residues, leverage strong electrostatic interactions with anionic cell surface glycosaminoglycans and phospholipids that can initiate clathrin-mediated endocytosis or macropinocytosis pathways, although in some contexts, they may undergo direct translocation.

Amphipathic peptides, characterized by spatial segregation of hydrophobic and hydrophilic domains, can insert into lipid bilayers to perturb membrane integrity, enabling direct penetration or enhancing endosomal escape via transient pore formation. Cyclic peptides, whose conformational rigidity and reduced proteolytic susceptibility confer high stability and receptor affinity, generally depend on ligand-specific receptor-mediated endocytosis, often with improved targeting precision.

Stimuli-responsive sequences, such as pH-, redox-, or enzyme-cleavable motifs, are designed to remain functionally inactive in circulation but become activated in the acidic microenvironment of endosomes, the reductive milieu of the cytosol, or in the presence of disease-associated proteases, thereby facilitating spatiotemporally controlled cargo release. Consequently, the intracellular trafficking routes and release mechanisms can be directly correlated with the physicochemical attributes and functional motifs, establishing a clear structure–function paradigm for the rational design of peptide-based delivery systems [41,42].

### 4.2. Classification of Peptides in PINPs

#### 4.2.1. Targeting Ligands

Targeting ligands play a crucial role in the design of peptide-functionalized MNPs for precision drug delivery. In a study by Kumar et al. [43], AuNPs were functionalized with the CRGDK peptide, explicitly targeting the neuropilin-1 (NRP-1) receptor, commonly overexpressed on the surface of tumor vasculature and specific cancer cells. CRGDK was easily conjugated to metal surfaces like gold via thiol chemistry, typically through cysteine residues, ensuring stable and oriented attachment. The high binding affinity of such peptide ligands to tumor-associated receptors facilitates receptor-mediated endocytosis, leading to enhanced accumulation within tumor microenvironments. This ligand-guided approach enhances the selective uptake of nanoparticles by tumor tissues, minimizing off-target distribution and associated toxicity. The targeted AuNPs were conjugated with a pro-apoptotic therapeutic peptide, PMI, which reactivates the p53 tumor suppressor pathway. By combining therapeutic and targeting functionalities, this dual-functional nanoparticle system significantly improved intracellular delivery in cancer models compared to non-targeted systems, and the targeted AuNPs exhibited enhanced therapeutic efficacy, attributed to both the precision delivery of the therapeutic payload and the tumor-homing ability of the peptide. This study demonstrated the importance of rationally selected peptide ligands in designing next-generation nanomedicines for targeted cancer therapy.

#### 4.2.2. Capping and Stabilizing Agents

Using peptides to cap and stabilize MNPs has emerged as a key strategy in drug delivery, offering enhanced control over nanoparticle size, surface chemistry, biocompatibility, and target specificity [25]. MNPs such as AuNPs, AgNPs, and Fe_3_O_4_ NPs possess unique physicochemical properties that are highly valuable for imaging, therapy, and responsive drug release [44]. However, their biomedical application depends heavily on their stability in biological environments, which can be effectively addressed by peptide functionalization.

Peptides as capping agents play a dual role: they stabilize nanoparticles by preventing aggregation and confer biofunctionality by offering recognition motifs for targeting or cell entry [26,27]. The presence of amino acids like cysteine, histidine, lysine, and glutamate allows peptides to bind metal ions or surfaces through thiol, amine, or carboxyl groups, forming strong coordinative bonds. This anchoring controls the nucleation and growth of MNPs and creates a protective layer that resists enzymatic degradation and opsonization [28,29]. For example, cysteine-containing peptides can effectively cap AuNPs via thiol–gold interactions, forming stable colloids under physiological conditions [45].

In the context of drug delivery, capping agents serve additional functions beyond stabilization. They can be engineered to bind therapeutic agents via covalent or non-covalent interactions and may contain targeting moieties (e.g., peptides or antibodies) to direct nanoparticles to specific disease sites. Peptides, for instance, serve both as capping agents and biological ligands, enabling receptor-mediated delivery while maintaining nanoparticle stability [23,46].

Additionally, peptide capping enhances tumor specificity and improves nanoparticle stability and biocompatibility while minimizing rapid clearance by the mononuclear phagocyte system (MPS), which preferentially removes particles larger than ~200 nm [47]. These features make peptide-MNPs promising platforms for targeted cancer therapy.

#### 4.2.3. Reducing Agents

MNPs such as AuNPs, AgNPs, and Fe_3_O_4_ NPs offer valuable properties for drug delivery, including high surface area, tunable size, and unique optical or magnetic characteristics. However, to ensure their safe and effective use in biomedical applications, it is essential to control their synthesis and surface properties [43]. This is achieved through reducing agents, which convert metal ions into zero-valent metal atoms, and capping agents, which stabilize the resulting nanoparticles by preventing aggregation and providing biocompatibility.

Reducing agents are critical during the synthesis phase, as they determine the nucleation and growth of nanoparticles. Traditional reducing agents such as sodium borohydride (NaBH_4_) and hydrazine hydrate are effective but often produce toxic byproducts, limiting their suitability for biomedical applications [5]. As a result, greener alternatives such as ascorbic acid, citrate, plant extracts, and amino acids are increasingly used. For example, citrate functions both as a mild reducing agent and a weak capping agent in the classic Turkevich method for synthesizing AuNPs, producing nanoparticles with excellent dispersibility [48]. Biological reducing agents offer the added advantage of biocompatibility and environmental safety, making them attractive for clinical translation.

Moreover, the nature of the capping and reducing agents influences the drug release profile, biodistribution, and toxicity of MNP-based drug carriers. Stimuli-responsive capping agents that degrade under specific pH, redox, or enzymatic conditions have been developed for controlled and site-specific drug release, especially in tumor microenvironments [49,50]. Table 4 summarizes the functional roles of peptides in PINPs with examples.

## 5. Synthesis of PINPs

The synthesis of PINPs involves integrating the functional diversity of peptides with the unique physicochemical properties of MNPs. Peptides serve multiple roles in the synthesis process: reducing agents, capping/stabilizing ligands, and structure-directing templates, offering an environmentally friendly and biocompatible approach to nanoparticle formation [25,26,27,28].

### 5.1. Peptide-Mediated Synthesis

Peptide-mediated synthesis of PINPs is an emerging, eco-friendly approach that utilizes peptides’ structural and chemical versatility to reduce, nucleate, and stabilize metallic nanostructures. Unlike traditional chemical methods that often require harsh reducing agents and surfactants, peptide-based synthesis offers precise control over nanoparticle size, morphology, and surface functionality under mild, aqueous, and biocompatible conditions [55,56].

Peptides serve multiple roles in nanoparticle synthesis. Depending on their amino acid composition and sequence, peptides can act as reducing agents, nucleation templates, and capping or stabilizing agents [48,57]. Amino acids such as tyrosine, cysteine, histidine, lysine, and tryptophan play critical roles due to their redox activity and strong affinity for metal ions. For instance, through their phenolic hydroxyl groups, tyrosine residues can reduce gold ions (Au^3+^) to elemental gold (Au^0^). At the same time, cysteine provides thiol groups that tightly bind to metal surfaces, aiding in particle stabilization [58,59].

A widely studied example is the synthesis of AuNPs using peptides derived from or inspired by biological motifs. Short peptides containing cysteine residues can reduce gold ions and simultaneously bind to the growing gold surface to limit particle growth and aggregation [60]. This dual role ensures the formation of well-dispersed, monodisperse nanoparticles with tunable sizes and morphologies.

**Figure 4:**
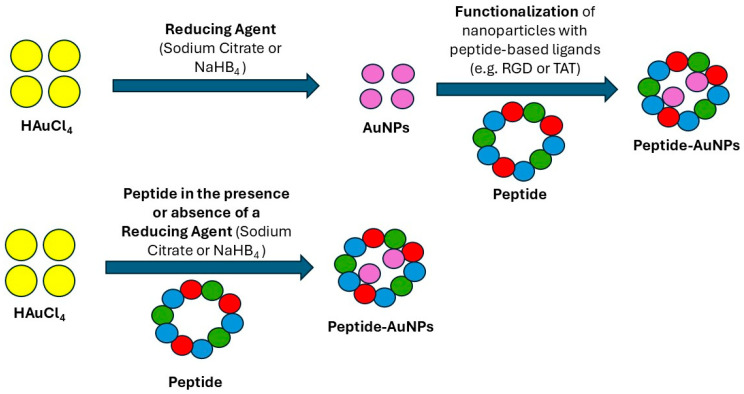
Synthesis of peptide–gold nanoparticles through different methods.

Furthermore, self-assembling peptides can template the formation of ordered metal nanostructures, including nanowires and nanotubes, by organizing metal ions along their supramolecular scaffolds, which helps construct bioelectronic and sensing devices [61]. Figure 4 summarizes common PINP synthesis approaches.

#### 5.1.1. One-Pot Green Synthesis

The one-pot green synthesis of PINPs represents a sustainable and biocompatible approach to fabricating hybrid nanostructures suitable for biomedical applications such as drug delivery, imaging, and biosensing. In contrast to conventional methods that often rely on toxic reducing agents (e.g., sodium borohydride) and synthetic stabilizers (e.g., surfactants), green synthesis utilizes naturally derived or biologically inspired molecules, including peptides, to both reduce metal ions and stabilize the formed nanoparticles in a single reaction step [25,61,62].

In a one-pot system, the entire synthesis occurs in a single vessel under mild conditions (aqueous solvent, ambient or slightly elevated temperature, neutral or near-neutral pH), with peptides simultaneously acting as reducing agents, nucleation templates, and capping ligands. This eliminates the need for additional purification steps and aligns with principles of green chemistry by minimizing hazardous byproducts, reducing energy consumption, and ensuring biocompatibility of the final product [63,64].

The reaction conditions in one-pot peptide-mediated synthesis are highly tunable. Variables such as pH, temperature, peptide concentration, and metal ion concentration can influence the nucleation rate, particle size, shape, and surface charge. For instance, lower pH may enhance metal ion solubility but reduce peptide binding efficiency, while higher peptide concentrations typically yield smaller nanoparticles due to rapid surface passivation [65,66,67]. The simplicity of the one-pot method also supports scalability and reproducibility, which are essential for translating PINPs to clinical or industrial settings.

An additional advantage of this technique is the functional versatility of the peptides. Since peptides can be synthetically tailored, sequences can be designed to include cell-penetrating motifs (e.g., TAT), targeting ligands (e.g., RGD), or stimulus-responsive elements (e.g., matrix metalloproteinase-cleavable linkers). As a result, the synthesized PINPs are inherently functional and may not require additional surface modifications for biomedical applications [68,69,70,71].

Thus, peptide-mediated synthesis of PINPs provides a versatile, green, and tunable strategy for developing advanced nanomaterials with biomedical relevance. The ability to rationally design peptides for controlled nucleation, stabilization, and targeting opens new avenues for precision nanomedicine and materials science. One-pot green synthesis using peptides provides a simple, eco-friendly, and customizable route to generate PINPs with enhanced biocompatibility and application-ready functionality. This strategy holds immense potential for the development of next-generation nanotherapeutics.

#### 5.1.2. Reducing and Stabilizing Activities of Peptides in the Synthesis of PINPs

Due to their inherent biocompatibility and functional diversity, peptides have emerged as powerful biomolecular tools in the green synthesis of MNPs. As described above, they serve a dual role in the synthesis process: acting as reducing agents to convert metal ions into nanoparticles and stabilizing (capping) agents to prevent aggregation and maintain nanoparticle integrity [72]. These properties are particularly advantageous for developing PINPs as nanocarriers for drug delivery, where safety, surface functionality, and biological compatibility are essential.

Peptides derive their reducing and stabilizing capabilities from specific amino acid side chains. Tryptophan, for example, contains an electron-rich indole ring that can donate electrons to reduce metal ions like Au^3+^ under mild aqueous conditions [73,74]. Its aromatic structure also participates in π–π interactions, aiding nanoparticle stabilization and surface passivation. In drug delivery, such nanoparticles can serve as carriers with high surface area and tunable binding capacity for hydrophobic drugs or targeting ligands. Another example is the synthesis of AuNPs using cell-penetrating peptides, achieving high payload and uptake efficiency in HeLa cells [75].

Tyrosine plays a similar role through its phenolic hydroxyl group, which undergoes oxidation during metal ion reduction. Tyrosine-rich peptides have been successfully used to produce biocompatible AuNPs suitable for delivering anticancer agents or contrast agents in tumor imaging [76]. These nanoparticles often exhibit pH stability and good dispersibility in physiological media, which is essential for intravenous administration [77,78].

Cysteine provides thiol groups that form strong covalent bonds with metals, ensuring long-term colloidal stability. Peptides containing cysteine are particularly well-suited for drug delivery because the stabilized surface allows for the conjugation of drugs, polyethylene glycol (PEG), or targeting ligands without compromising nanoparticle integrity [79,80,81].

Histidine and lysine, with their imidazole and amine side chains, respectively, coordinate metal ions and offer electrostatic interactions that guide nanoparticle nucleation and growth. Additionally, their positive charge at physiological pH enhances cellular uptake, a critical parameter for effective intracellular drug delivery [82].

By leveraging the redox and coordinating capabilities of amino acid residues such as tryptophan, tyrosine, cysteine, and histidine, peptides serve as eco-friendly and effective agents in the synthesis of PINPs. These peptide-stabilized nanocarriers offer enhanced biocompatibility, targeted delivery potential, and functional versatility, making them a promising platform for next-generation drug delivery systems.

Moreover, the stabilizing peptides also help control the release profile of loaded drugs. For example, MNPs capped with amphiphilic peptides can encapsulate hydrophobic drugs and release them in response to environmental stimuli like pH or enzymes found in tumor microenvironments [83,84]. This contributes to improved drug solubility, reduced systemic toxicity, and enhanced accumulation at disease sites via the EPR effect.

#### 5.1.3. Conjugation Methods Through Surface Functionalization and Self-Assembly

The conjugation of peptides to MNPs is a powerful strategy to enhance nanomaterials’ functionality, biocompatibility, and specificity for biomedical applications, especially drug delivery. Unlike one-pot green synthesis, where peptides act as reducing and stabilizing agents, conjugation-based synthesis involves post-synthetic modification of pre-formed MNPs with functional peptides. This approach offers precise control over peptide orientation, density, and bioactivity, making it ideal for constructing targeted, stimulus-responsive, and multifunctional nanocarriers.

The synthesis begins with preparing bare or ligand-stabilized MNPs, commonly composed of gold (AuNPs), silver (AgNPs), iron oxide, or other metallic cores. These nanoparticles typically present reactive surface groups such as hydroxyl, carboxyl, or amine functionalities, or they are modified with stabilizing ligands like citrate or polyethylene glycol (PEG) to provide colloidal stability and facilitate subsequent conjugation reactions [60]. Self-assembling peptide scaffolds can also template ordered metal nanostructures (nanowires, nanotubes) by guiding metal deposition along their fibrils [85].

Peptide conjugation can be achieved through several chemical strategies:

(a) Thiol–Gold Chemistry: Peptides containing cysteine residues are directly conjugated to AuNP surfaces via strong Au–S bonds, forming stable monolayers. This method is widely used due to its simplicity and high binding affinity [86].
(b) EDC/NHS Coupling: Carboxyl-functionalized nanoparticles can be activated using 1-ethyl-3-(3-dimethylaminopropyl)carbodiimide (EDC) and N-hydroxysuccinimide (NHS) (Figure 1) to form an active ester intermediate that reacts with amine groups on peptides, forming stable amide bonds. This allows for site-specific attachment of peptides without disrupting their bioactivity [87].
(c) Click Chemistry: Copper-catalyzed azide-alkyne cycloaddition (CuAAC) provides high-efficiency conjugation with orthogonal selectivity, making it suitable for dual functionalization (Figure 1) or creating PINPs with controlled spacing [88].
(d) Physical Adsorption: Electrostatic or hydrophobic interactions may also drive non-covalent peptide adsorption onto nanoparticle surfaces. While simpler, this method offers weaker binding and may suffer from desorption under physiological conditions [25].

Moreover, metal nanoparticles could be generated under various circumstances based on the chemical method. In Table 5, several methods for synthesizing metal nanoparticles are compared and elaborated on.

## 6. Drug Loading Capacity and Surface Area

PINPs with smaller diameters offer higher surface area-to-volume ratios, enhancing their capacity for surface drug loading or functionalization with targeting ligands. Incorporating peptides further augments the drug loading efficiency by providing additional binding sites through their charged or hydrophobic residues.

This modular structure allows for both covalent and non-covalent loading strategies, enabling the delivery of diverse therapeutic cargos such as anticancer agents (e.g., doxorubicin), antiviral drugs (e.g., emtricitabine/lamivudine), or signaling peptides (e.g., phosphotyrosine-containing peptides) [25,26,27,40]. As a result, PINPs combine structural robustness with multifunctionality, making them highly versatile and efficient nanocarriers for drug delivery [25,26,27,28].

## 7. Pharmacokinetics: Clearance and Circulation Time

### 7.1. Size-Dependent Clearance Mechanisms

Clearance and circulation time are other critical criteria. The pharmacokinetics of PINPs are heavily influenced by their size, surface chemistry, and structural design. Metal cores such as gold or iron oxide, when functionalized with peptides, typically fall within the optimal size range of 10–100 nm, large enough to avoid rapid renal clearance (which dominates for particles < 6 nm) yet small enough to evade uptake by the mononuclear phagocyte system (MPS) [24,94]. The choice of peptide affects not only targeting but also biodistribution and clearance [95,96,97].

### 7.2. Cyclic Nature Properties

Cyclic peptides and amphiphilic sequences tend to improve nanoparticle stability and reduce premature aggregation, both of which contribute to favorable circulation profiles. The cyclic configuration of peptides, created through covalent bonding between *N*- and *C*-termini or side chains, results in a structurally rigid formation. The cyclic nature maintains its functional integrity and prolongs circulation time. In addition, the cyclic nature enhances stability during endocytosis upon internalization into cells. This feature will decrease metabolic degradation, leading to a longer half-life and sustained release effect. This enhanced stability may be attributed to their unique cyclic structure, which lacks free *N*- or *C*-termini for enzymatic degradation, in contrast to their linear counterparts that are more susceptible to enzymatic cleavage due to the presence of these terminal sites.

Moreover, cyclic peptides exhibit selective binding capabilities by minimizing unwanted shape changes while increasing the surface area available for interactions with target receptors. This stability enhances binding affinity and specificity. Cyclic peptides can be tailored to recognize overexpressed receptors on cancerous or inflamed tissues, making them particularly suitable for targeted drug delivery. Together, these characteristics, structural stability, metabolic resistance, and high selectivity, make cyclic peptides a powerful platform for therapeutic applications [52].

In addition, cyclic peptides often have high binding affinity and structural stability due to their constrained conformation. This rigidity improves resistance to proteolysis and can be engineered to recognize specific receptors on target cells (e.g., RGD cyclic peptides binding integrins). However, once the peptide-based nanoparticles are inside the cells, they can become trapped in endosomes. Many cyclic peptides suffer from conformational flexibility or from the amphipathic character that is required to disrupt endosomal membranes. At this stage, amphipathic motifs combine hydrophobic and hydrophilic regions, enabling insertion into lipid bilayers and destabilization of endosomal membranes. This characteristic facilitates endosomal escape, complementing the targeting ability of cyclic peptides [98].

For instance, cyclic KW_5_-functionalized gold nanoparticles demonstrated enhanced cellular uptake of doxorubicin compared to linear analogs [25], highlighting how peptide design could impact in vivo performance. Thus, rational engineering of PINPs offers control over clearance pathways and circulatory behavior, which is crucial for achieving effective and targeted drug delivery.

### 7.3. Size-Dependent Toxicity

Size-dependent toxicity and immune response are significant elements in drug delivery. The biological safety of PINPs is tightly linked to their physicochemical properties, particularly particle size [99,100,101]. Size determines biodistribution and clearance and plays a critical role in cytotoxicity and immune recognition.

PINPs (e.g., gold, silver, or iron oxide cores) in the optimal size range of 10–100 nm generally exhibits low toxicity and minimal immune activation due to their ability to evade rapid uptake by immune cells and avoid aggregation. However, particles smaller than 10 nm may penetrate deeply into cells and organelles, potentially interfering with mitochondrial or nuclear function. In comparison, particles larger than ~100–150 nm are more likely to be recognized by macrophages, leading to accelerated clearance and immune stimulation [102,103,104].

The presence of peptides on the nanoparticle surface modulates the immune response by altering protein corona formation and cellular interactions [105]. Cationic or amphiphilic peptides may increase membrane permeability or interact with negatively charged cell surfaces, enhancing delivery and raising concerns about hemolysis or inflammatory signaling if not carefully designed [106,107].

PEGylation or using zwitterionic peptide motifs can reduce immunogenicity by minimizing nonspecific protein adsorption and complement activation [108]. In one study, cyclic peptide-capped gold AuNPs showed excellent biocompatibility and negligible toxicity, demonstrating that peptide structure and density can influence efficacy and safety profiles [25,26]. Thus, optimizing the size and surface peptide architecture of MNPs is essential for minimizing toxicity and achieving favorable immune tolerance in therapeutic applications.

### 7.4. Incorporation of Non-Natural Amino Acids into the Structure of Peptides

One of the effective strategies to enhance peptide stability against enzymatic degradation involves replacing natural amino acids with non-natural or unnatural analogues. These modifications often include the incorporation of *d*-amino acids, *N*-methylated amino acids, β-amino acids, and peptidomimetic structures. For instance, substituting L-amino acids with their *d*-counterparts at protease cleavage sites significantly reduces susceptibility to enzymatic hydrolysis, thereby extending peptide half-life. *N*-Methylation of the peptide backbone constrains conformational flexibility, which not only improves protease resistance but can also enhance membrane permeability. Additionally, β-amino acids residues increase metabolic stability by evading recognition by proteolytic enzymes, often resulting in improved pharmacokinetic properties [109].

## 8. Cellular Uptake Mechanisms of Peptide-INPs (PINPs)

**Figure 5:**
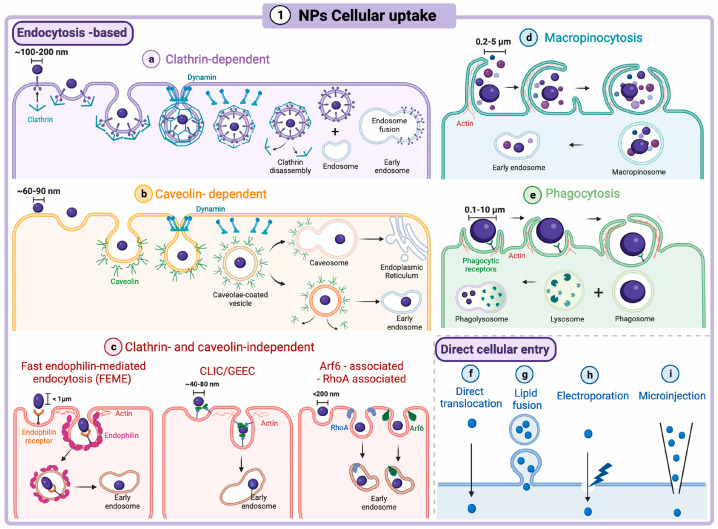
Schematic representation showing the mechanisms of nanoparticle cellular internalization divided into endocytosis–based mechanisms (**left**), which include (**a**) clathrin-dependent, (**b**) caveolin-dependent, (**c**) clathrin- and caveolin-independent, (**d**) phagocytosis, and (**e**) micropinocytosis; and direct cellular entry (**right**), which includes (**f**) direct translocation, (**g**) lipid fusion, (**h**) electroporation, and (**i**) microinjection. Reprinted with permission [110]. Copyright 2023 Science Direct.

The cell membrane serves as a selective barrier to protect the intracellular components from the external environment while regulating the transportation of substances into the cell. Nanoparticles take advantage of various endocytosis-dependent mechanisms to deliver molecular cargos intracellularly, including phagocytosis, Clathrin-mediated endocytosis (CME), caveolae-dependent endocytosis, Clathrin/caveolae independent endocytosis, and macropinocytosis. Additionally, other internalization mechanisms have also been reported, such as passive diffusion, hole formation, direct microinjection, and electroporation [55,109]. A schematic representation of different mechanistic pathways for nanoparticles to enter cells is illustrated in Figure 5.

Mechanistically, amphipathic peptides enhance endosomal escape primarily through the interplay of their hydrophobic and cationic domains. The hydrophobic segments insert into the lipid bilayer of the endosomal membrane, disrupting lipid packing and increasing membrane permeability. Simultaneously, the positively charged residues (e.g., lysine, arginine) electrostatically interact with negatively charged phospholipids and glycosaminoglycans, inducing membrane destabilization. This dual action facilitates transient pore formation or complete membrane disruption, enabling the nanoparticle–cargo complex to translocate into the cytosol before lysosomal degradation occurs. The degree of hydrophobicity and charge density critically influences this process; higher hydrophobicity promotes deeper membrane insertion, while optimal charge density maximizes electrostatic interactions without causing irreversible membrane damage that could trigger cytotoxicity.

The relationship between a peptide’s hydrophobic moment (amphipathic character) and its endosomal escape efficiency has been of considerable interest. Direct statistical correlations in the literature are limited, but available evidence suggests a positive trend: peptides with greater hydrophobicity or amphipathicity often exhibit improved endosomal escape. For instance, Allen et al. demonstrated that augmenting an arginine-rich cell-penetrating peptide with hydrophobic moieties enhanced its ability to deliver a fluorescent payload into the cytosol (indicating efficient endosomal membrane disruption) [111]. In their systematic study, CPP variants with low hydrophobic moment failed to release cargo, whereas those with increasingly hydrophobic additions achieved robust endosomal escape and cytosolic delivery of the payload. This implies that a higher hydrophobic moment, typically meaning that a peptide has a well-defined amphipathic helix or hydrophobic face, correlates with greater membrane lytic activity at the endosomal membrane, facilitating escape. That said, no comprehensive meta-analysis has quantitatively linked hydrophobic moment values to escape efficiency across diverse peptides; the trend is more qualitative. This literature review did not reveal a universal metric or formula predicting endosomal escape from hydrophobic moment alone, likely because other factors (peptide length, charge, pH-responsiveness, etc.) also play important roles. Nevertheless, the qualitative trend is that increasing amphipathic hydrophobic character in endosomolytic peptides tends to improve their endosomal escape performance.

In terms of cellular uptake mechanisms, Shirazi et al. demonstrated that AuNPs functionalized with cyclic [KW]_5_ peptides internalize far more efficiently into cancer cells (e.g., CCRF-CEM and SK-OV-3) than their linear counterparts or free peptides [25]. Flow cytometry investigations revealed an approximate 12–13-fold enhancement in the uptake of fluorescent cargos (FTC-lamivudine and the phosphopeptide, GpYEEI) when using cyclic [KW]_5_-AuNPs compared to linear (KW)_5_-AuNPs. Confocal imaging showed distinct intracellular localization patterns: cyclic constructs delivered cargo predominantly into the nucleus within 1 h, whereas linear peptide-AuNPs confined the payload to the cytoplasm [25]. Similar investigations were conducted with SeNPs and GdNPs [27,28]. All observations suggest that nanoparticle morphology and peptide conformation, for instance, sponge-like for cyclic versus spherical for linear, critically influence cellular membrane interactions and subsequently their cellular uptake.

Although these studies did not dissect exact entry routes (e.g., clathrin, caveolin, or macropinocytosis), they strongly implied that the cyclic peptide-INPs harness CPP-like mechanisms akin to endocytosis-mediated uptake. Their high density of lysine/tryptophan residues likely promotes electrostatic binding to cell-surface glycoproteins, followed by internalization through energy-dependent processes typical of cell-penetrating peptides. The fact that cyclic peptide-capped AuNPs deliver cargos into the nucleus also hints at possible endosomal escape and nuclear targeting via nuclear pore complexes, enabled by the peptide’s amphipathic and positively charged nature. Thus, peptide–AuNPs enhance passive diffusion through membrane interactions and may actively engage endocytic pathways to achieve efficient cytosolic and nuclear delivery [26,27,28,29].

## 9. Delivery of Drugs Using PINPs

### 9.1. Targeted Delivery

Targeted drug delivery is a strategy that enhances the therapeutic efficacy and minimizes the systemic toxicity of chemotherapeutic agents by directing them to specific tissues or cells. Among the various nanocarriers developed for this purpose, PINPs have emerged as promising platforms due to their tunable physicochemical properties, biocompatibility, and functional versatility. Peptides deliberate targeting specificity, while the metal core can offer imaging, therapeutic, or structural advantages. Together, they enable precise delivery of drugs to disease sites, particularly in cancer therapy.

The metal core enhances the multifunctionality of these systems. For example, AuNPs provide excellent drug-loading capacity and photothermal conversion efficiency, enabling combined chemo–photothermal therapy [112,113]. In one study, doxorubicin-loaded AuNPs functionalized with RGD peptides showed increased tumor accumulation and reduced off-target toxicity compared to free drug formulations [114]. Iron oxide nanoparticles, on the other hand, allow for magnetic targeting and MRI imaging, and when conjugated with tumor-homing peptides such as RGD and chlorotoxin (CTX), enable image-guided delivery [115,116].

Beyond targeting and release, peptides also improve biocompatibility and circulation stability, reducing opsonization and clearance by the mononuclear phagocyte system. Hydrophilic residues can help stabilize PINPs in physiological conditions, extending blood circulation time and improving therapeutic outcomes. For instance, polyethylene glycol-conjugated gold nanoparticles create a hydrophilic “stealth” layer that reduces adsorption, decreasing macrophage recognition and clearance and extending blood circulation time [117,118].

PINPs offer a robust and modular platform for targeted drug delivery by combining the recognition capabilities of peptides with the functional advantages of metal nanocores. These hybrid systems enhance therapeutic precision, reduce side effects, and open avenues for personalized and image-guided medicine.

Several studies have quantitatively compared the potency of drug-loaded PINPs with free drug using IC_50_ or MIC_50_ metrics, consistently showing improved therapeutic efficacy. For example, copper-coated erythromycin-loaded nanocapsules exhibited a reduction in MIC_50_ relative to both free and uncoated drug, demonstrating synergistic antibacterial effects from the copper layer [119]. Similarly, doxorubicin- and paclitaxel-loaded superparamagnetic iron oxide nanoparticles (SPIONs) showed significantly lower IC_50_ values against A2780 and OVCAR-3 ovarian cancer cells compared to free drugs, attributed to EPR and prolonged intracellular retention [120]. In another example, black pomegranate peel extract encapsulated in chitosan-coated magnetic nanoparticles achieved markedly lower IC_50_ values against MDA-MB-231 and 4T1 breast cancer cells than the free extract while maintaining biocompatibility with normal cells [121]. Collectively, these findings indicate that incorporation of drugs into peptide- or polymer–metal nanoparticle systems can significantly reduce IC_50_/MIC_50_ values compared to free drug, enhancing potency through improved delivery, cellular uptake, and in some cases, metal-assisted therapeutic effects.

### 9.2. Stimuli-Responsive Systems

Stimuli-responsive drug delivery systems are engineered to release therapeutic agents in response to specific biological or physicochemical triggers at the disease site, thereby improving therapeutic efficacy while minimizing off-target effects. PINPs represent a unique class of stimuli-responsive systems due to the integration of functional peptides with the optical, catalytic, or magnetic properties of metal nanocores. These hybrid nanostructures can be precisely designed to respond to internal stimuli (e.g., pH, redox, enzymes) or external stimuli (e.g., light, temperature, magnetic fields), enabling spatiotemporally controlled drug release.

#### 9.2.1. pH-Responsive Systems

pH-sensitive peptides can undergo conformational changes in the acidic tumor microenvironment, triggering drug release. The tumor microenvironment is slightly acidic (pH ~6.5), contrasting with the physiological pH of 7.4. PINPs can be functionalized with histidine-rich peptides that are protonated under acidic conditions, leading to conformational changes or disruption of peptide–drug interactions, thereby triggering drug release [50]. AuNPs capped with pH-sensitive peptide sequences have shown enhanced doxorubicin release under acidic conditions mimicking tumor environments [122].

#### 9.2.2. Enzyme-Responsive Systems

Many tumors overexpress specific proteolytic enzymes such as matrix metalloproteinases (MMPs) or cathepsins. Peptides containing MMP-cleavable linkers can be conjugated to MNPs for site-specific activation. Upon exposure to MMPs, the peptide cleaves, resulting in drug release or nanoparticle disassembly [123,124,125]. This approach enhances target selectivity and reduces systemic toxicity.

#### 9.2.3. Redox-Responsive Systems

Glutathione (GSH) is present at higher concentrations in the intracellular environment, especially in cancer cells. Disulfide-bridged peptides on PINPs are cleaved in this reductive environment, leading to intracellular drug release. For example, peptide-modified AgNPs incorporating disulfide bonds demonstrated GSH-triggered release of paclitaxel within tumor cells [126,127].

#### 9.2.4. Light and Heat Responsiveness

Gold and silver nanoparticles exhibit strong surface plasmon resonance (SPR), enabling them to convert light into heat upon near-infrared (NIR) irradiation. Peptides can be used to stabilize these MNPs and carry drugs that are released through photothermal disruption of peptide–drug interactions. In one study, RGD-peptide modified AuNPs loaded with doxorubicin released their cargo upon NIR stimulation, achieving combined photothermal and chemotherapeutic effects [128,129].

#### 9.2.5. Magnetic Responsiveness

Iron oxide nanoparticles (Fe_3_O_4_), when functionalized with targeting peptides, can be directed to tumor sites using external magnetic fields. Combined with enzyme- or pH-responsive linkers, these systems allow for magnetically guided, stimuli-triggered drug release, with added MRI contrast capabilities [130].

By integrating smart peptide designs with metal nanoparticle platforms, stimuli-responsive PINPs enable on-demand drug release, enhanced target specificity, and improved treatment outcomes. Their adaptability to internal and external stimuli positions them as powerful tools for next-generation precision nanomedicine.

## 10. Advantages of PINPs

Several features enable PINPs to serve as multifunctional platforms for targeted, responsive, and traceable drug delivery systems. PINPs engineered with amphiphilic peptides like SAP have been shown to deliver cargo while maintaining low cytotoxicity and avoiding aggregation even under physiological ionic conditions. Furthermore, PKCδ-targeting peptide drugs were effectively delivered using peptide–AuNP hybrids, demonstrating potential in acute lung injury treatment models [75].

### 10.1. Biocompatibility and Biodegradability

Biocompatibility and biodegradability are essential characteristics for any nanocarrier intended for therapeutic use, as they directly affect safety, systemic clearance, immune response, and eventual clinical translation. PINPs offer a compelling platform due to their hybrid composition, where peptides provide biofunctionality and metal cores offer structural and therapeutic advantages. The unique synergy of these components enables the design of functional but well-tolerated and biodegradable nanostructures in biological systems.

Additionally, peptides can serve as biocompatible capping and stabilizing agents, improving nanoparticle dispersion and circulation time. The metal core contributes structural stability, enables controlled drug loading and release, and often offers imaging or therapeutic functionalities, such as photothermal or magnetic properties [131]. Together, these features allow PINPs to serve as multifunctional platforms for targeted, responsive, and traceable drug delivery systems.

Peptides, by their nature, are biodegradable and generally non-toxic. They can be enzymatically cleaved by proteases into harmless amino acid fragments, making them attractive candidates for nanoparticle surface functionalization or stabilization. Moreover, peptides can be engineered to incorporate biodegradable linkers, stimuli-sensitive sequences, and biologically inert motifs to minimize immune recognition and prolong systemic circulation [132]. Their ability to mimic endogenous sequences also reduces the likelihood of adverse immune responses, supporting long-term biocompatibility.

Biocompatibility assessments of PINPs have shown low cytotoxicity in various in vitro and in vivo studies. The metal component of PINPs [133], such as gold (Au), silver (Ag), iron oxide (Fe_3_O_4_), selenium (Se), or gadolinium (Gd), varies in biodegradability. Although AuNPs are not inherently biodegradable, they are considered inert and highly biocompatible at low concentrations. Their clearance is often dependent on particle size, surface charge, and coating, with smaller particles being renal-clearable.

Iron oxide nanoparticles, conversely, are both biocompatible and biodegradable, as iron ions released from degradation can be incorporated into the body’s natural iron metabolism pathways [134]. Iron oxide nanoparticles conjugated with tumor-homing peptides exhibited efficient tumor targeting with minimal liver or kidney toxicity [135].

SeNPs, stabilized by peptides, have demonstrated low toxicity and favorable antioxidant and antitumor properties, with partial biodegradability via selenoprotein metabolism [136]. GdNPs can offer contrast enhancement for imaging but require peptide coatings to reduce toxicity and control biodistribution due to concerns over free Gd ion release [137].

Peptide-capped AgNPs demonstrated negligible toxicity to normal cells while exhibiting selective cytotoxicity against cancer cells, indicating targeted delivery and reduced off-target effects [138].

In summary, the biodegradable nature of peptides and the tailorable biocompatibility of metal cores make PINPs strong candidates for safe and effective drug delivery. Rational selection of both peptide sequence and metal type, combined with appropriate surface engineering, enables the development of functionally superior and biologically acceptable nanocarriers.

### 10.2. Specific Targeting Capabilities

A significant challenge in drug delivery is achieving precise delivery of therapeutic agents to diseased tissues while sparing healthy cells. PINPs offer unique advantages in this regard, as peptides can be engineered to bind specific cellular receptors. At the same time, the MNP core provides a versatile platform for drug loading, imaging, and therapy. This targeted approach enhances drug accumulation at the desired site, improves therapeutic efficacy, and reduces systemic toxicity.

One example includes peptides targeting HER2 (human epidermal growth factor receptor 2), EGFR (epidermal growth factor receptor), and VEGFR (vascular endothelial growth factor receptor), which are often overexpressed in various cancer types. AuNPs and AgNPs conjugated with HER2-binding peptides have shown selective uptake by HER2-positive breast cancer cells, allowing for targeted therapy and real-time imaging [139,140].

Beyond cancer, peptides have been used to target infectious agents. For example, AMPs can direct MNPs to bacterial membranes, enabling localized antibacterial effects. Similarly, brain-targeting peptides (e.g., transferrin receptor-binding peptides) can facilitate crossing the blood–brain barrier, enabling drug delivery for neurological disorders [141].

The small size and surface tunability of PINPs also contribute to their ability to penetrate deep into tumors and accumulate via the EPR effect, with active targeting by peptides further refining selectivity. The metal core may also serve as a contrast agent (e.g., gold for CT imaging, iron oxide for MRI), enabling theranostic applications, simultaneous diagnosis, and therapy [142].

### 10.3. Versatile Functionalization

The multifunctionality of PINPs stems mainly from their ability to undergo precise and diverse functionalization strategies, enabling tailored applications in drug delivery, imaging, diagnostics, and therapy. The surface chemistry of MNPs (e.g., gold, silver, iron oxide) allows for stable conjugation with peptides through various interactions, including thiol–gold bonds, electrostatic forces, and covalent linkages, while peptides provide biorecognition, responsiveness, and targeting capabilities.

Moreover, multifunctional peptides can be designed to perform several roles simultaneously, such as targeting, drug binding, and self-assembly. For instance, amphipathic peptides enhance cellular uptake. RGD motifs specifically target integrins and, along with appropriate peptide sequences, respond to acidic pH, enabling pH-triggered drug release within the tumor microenvironment [122]. Such modular designs make PINPs versatile and adaptable for various biomedical uses.

The metal core further allows for the conjugation of non-peptidic molecules, such as fluorophores, drugs, or imaging agents, expanding the functionality of PINPs. For example, iron oxide nanoparticles functionalized with tumor-targeting peptides have been modified with fluorescent dyes for dual-mode imaging and therapy [50,142,143,144,145]. AuNPs functionalized with peptides and chemotherapeutic drugs in theranostics allow for combined targeted delivery, imaging, and photothermal therapy [84].

Notably, the modular nature of peptides allows for sequence customization, enabling the construction of nanoparticle surfaces with specific spatial arrangements, valencies, and cooperative functionalities. This level of control is critical for optimizing cellular uptake, endosomal escape, and therapeutic release, all of which are key factors in successful nanomedicine applications.

## 11. Challenges and Limitations

Despite the promise of PINPs in drug delivery and nanomedicine, several key challenges hinder their clinical translation. These include issues related to biological stability, toxicity and immunogenicity, scalable and reproducible synthesis, and regulatory and translational barriers. Addressing these limitations is essential to move PINPs from proof-of-concept studies to approved therapeutic platforms.

### 11.1. Stability in Biological Environments

When introduced into complex biological fluids, PINPs often face degradation, aggregation, or opsonization. Peptide coatings, while biocompatible, may desorb or degrade in serum due to enzymatic activity or ionic interactions, compromising nanoparticle integrity and function [146]. Moreover, the metal core, whether gold, silver, or iron oxide, may oxidize or undergo surface transformations, particularly under physiological pH and salt conditions. This leads to loss of targeting ability, premature drug release, or aggregation, reducing circulation time and therapeutic efficacy [147].

### 11.2. Immunogenicity and Toxicity Concerns

Though peptides are generally biodegradable and less immunogenic, their conjugation to MNPs can introduce immunostimulatory risks. Surface-exposed peptide epitopes may trigger immune recognition, especially with repeated administration. Meanwhile, metal cores like silver and gadolinium can release toxic ions or generate reactive oxygen species (ROS), contributing to cytotoxicity, organ accumulation, and inflammatory responses [137]. AuNPs, often considered inert, may still elicit biological effects depending on surface charge and size [148].

### 11.3. Scalability and Reproducibility

Producing PINPs at an industrial scale with batch-to-batch consistency remains a significant obstacle. Peptide synthesis, especially for sequences containing non-standard amino acids or disulfide bridges, can be costly and variable in purity. Another major reason could be their tendency to self-assemble into higher-order structures. Even minor differences in synthesis, purification, or handling can shift the equilibrium between monomeric and assembled states, leading to variations in particle size, morphology, surface properties, and stability. Such heterogeneity affects not only physicochemical characteristics but also biological performance, including cellular uptake and therapeutic efficacy.

Similarly, nanoparticle formation processes, often sensitive to pH, temperature, and reactant ratios, must be tightly controlled to ensure uniform size, surface functionalization, and drug loading efficiency. Even slight variations can alter biodistribution or targeting behavior, complicating preclinical evaluation [149]. All these elements together can reduce reproducibility across batches unless carefully controlled. Robust process standardization, stringent quality control, and detailed characterization of assembly parameters are essential to minimize variability and ensure consistent product quality [150].

### 11.4. Regulatory and Translational Barriers

PINPs exist at the intersection of biological and inorganic domains, presenting classification challenges to regulatory agencies. They do not fit neatly into existing categories for small molecules, biologics, or conventional nanomedicines, complicating safety evaluation and approval processes. Furthermore, the lack of standardized protocols for characterization, toxicity testing, and in vivo tracking creates additional hurdles. Long-term studies on bioaccumulation, metabolism, and excretion are often lacking, yet they are essential for clinical approval [151].

Overcoming these challenges will require interdisciplinary collaboration to optimize nanoparticle design, improve manufacturing processes, and establish clear regulatory frameworks. While PINPs offer a powerful tool for targeted and responsive drug delivery, addressing their biological complexity, safety, and scalability is essential for realizing their clinical potential.

### 11.5. In Vivo vs. In Vitro Outcomes

Overcoming the successful delivery of nanotherapeutics is often limited by biological barriers that prevent their selective accumulation at diseased sites, reducing their therapeutic efficacy in conditions such as cancer and inflammation. In vitro investigations are designed to understand the basics of the behavior in terms of stability, mechanism of action, and receptor binding affinity. However, successful in vitro outcomes do not necessarily reflect in vivo ones. Key challenges, including nonspecific distribution and poor site-specific retention, remain significant obstacles in in vivo experiments. To advance beyond these limitations, a fundamental redesign of nanoparticle systems is required, one that holistically considers the array of biological barriers encountered after intravenous administration. By addressing these challenges in a stepwise and integrated manner, it is possible to develop a new generation of nanocarriers capable of achieving precise, efficient, and clinically relevant drug delivery [95,152].

## 12. Design Rules for Next-Generation PINPs

Using peptide properties alone as predictive markers for the functionality of PINPs may not fully capture the complexity of these hybrid systems. The integration of metal components introduces additional physicochemical and biological interactions that must be considered independently from peptide-only systems.

To guide the fundamental redesign of PINPs for optimal efficacy, this review outlines key design principles that link peptide physicochemical properties, such as structural rigidity and amphipathicity, to functional intracellular delivery outcomes. These design rules aim to harness peptide attributes (e.g., linear vs. cyclic structure, cationic/amphipathic balance) to improve stages of delivery like cellular uptake, endosomal escape, and targeted release. Importantly, they build on insights gained from current studies and address the challenges identified above in developing next-generation peptide–nanoparticle conjugates.

As described above, Shirazi et al. [25] demonstrated that AuNPs functionalized with a cyclic [KW]_5_ peptide achieved ~12–13-fold higher cellular uptake of fluorescent cargo than analogous linear (KW)_5_–AuNPs. The cyclic [KW]_5_ construct delivered payloads predominantly into the nucleus within 1 h, whereas the linear peptide–AuNP confined the cargo in the cytoplasm. This dramatic uptake enhancement underscores how peptide conformation (cyclic vs. flexible linear) critically influences cell entry mechanisms and intracellular fate. Incorporating cyclization (through head-to-tail cyclization, disulfide bridges, etc.) as a design rule can improve nanoparticle stability in circulation and targeting precision. However, further research is required in this area before generalizing this rule to all PINPs.

Amphipathic peptides (often adopting α-helical or β-sheet structures upon membrane contact) can insert into lipid bilayers and destabilize them, thereby facilitating endosomal escape of nanoparticle cargo. Mechanistically, the hydrophobic face of an amphipathic peptide embeds into the endosomal membrane, disrupting lipid packing, while the cationic face interacts electrostatically with anionic phospholipid headgroups. The degree of hydrophobicity and charge density must be balanced: increasing hydrophobic content promotes deeper membrane insertion, but an overly high positive charge can cause irreversible membrane damage (and hence cytotoxicity) if not optimized. Design rules for the next generation of PINPs, therefore, should include tuning amphipathic sequences to achieve potent endosomolytic activity without excessive cell membrane disruption. In this context, inclusion of CPPs either as the primary capping ligand or as part of a multi-peptide formulation can markedly improve intracellular bioavailability of the nanoparticle’s payload. As a design guideline, it is important to ensure that such peptides have an optimal hydrophobic-cationic balance.

Another consideration is incorporating stimuli-responsive peptide elements as a crucial design rule to ensure endosomal escape of PINPs, thereby achieving cytosolic or nuclear delivery of cargo. For instance, pH-responsive amphipathicity, adding histidine-rich segments [50] that remain relatively innocuous at neutral pH but become positively charged in the acidic endosome, causes peptides to become amphipathically active in endosomes, leading to endosomal membrane destabilization and cargo escape. Table 6 compares some of the peptide classes that are currently used for designing PINPs, their application, and limitations.

An emerging design paradigm for next-generation PINP systems is to combine the strengths of different peptide classes within one construct to navigate all stages of delivery. As a general rule, multifunctional peptide coatings, incorporating modules for targeting, membrane penetration, and stimulus-responsive release, will define next-generation PMINP design. For example, inorganic nanoparticles coated with a cyclic targeting peptide plus a histidine-rich CPP could exhibit excellent tumor cell uptake and minimal off-target effects, highlighting the synergy of combining peptide functionalities. Optimal surface architecture (potentially using spacers or mixed monolayers of peptides) is therefore another design consideration.

In summary, next-generation PINPs should be rationally engineered with a balanced peptide toolkit, e.g., a robust targeting moiety, a cleavable or activatable CPP for endosomal escape, and perhaps a stealth component, to successfully navigate the series of biological barriers from bloodstream to cytosol.

## 13. Future Perspectives

PINPs have emerged as a versatile and promising class of nanomaterials for drug delivery, imaging, and diagnostics. As the field matures, future development will be driven by four synergistic pillars: advanced peptide engineering, multifunctional theranostic systems, artificial intelligence/machine learning (AI/ML)-guided design, and clinical translation strategies.

A deeper understanding of structure–activity relationships (SAR) enables the deliberate engineering of peptide ligands to enhance PINP performance. Several peptide engineering strategies have already proven effective in increasing stability, cellular uptake, and targeting specificity. These strategies include amino acid substitution, cyclization, length and charge optimization, and strategic conjugation and are instrumental in refining PINP performance. By implementing these modifications, researchers can create peptide–nanoparticle conjugates that are more stable, target-selective, and efficacious. For example, a PINP coated with a *d*-amino-acid cyclic peptide may resist degradation and function longer in vivo; if that peptide is also optimized in length and spacing for its receptor, the system will likely exhibit superior targeting and therapeutic index. Applying such SAR-driven design rules will ensure that peptide-functionalized nanocarriers are not only potent on the bench but also robust in the bloodstream and effective in the clinic.

By leveraging these design rules and engineering strategies, next-generation PINPs can be tailored to overcome biological barriers and maximize delivery efficiency. The insights from peptide SAR and the integration of multiple peptide functions lay a strong foundation for upcoming innovations. The following section discusses how these design principles fit into broader future perspectives and emerging trends (such as AI-driven design and clinical translation strategies) that will drive the field of PINP therapeutics.

Advanced peptide engineering will be central to enhancing specificity, stability, and functionality. Innovations such as non-natural amino acids, *d*-amino acid substitutions, cyclization, and stapled peptides can improve proteolytic resistance and receptor-binding affinity, expanding the therapeutic window of PINPs. Rational design and high-throughput screening, including phage display and combinatorial libraries, will allow precise control over peptide–receptor interactions and enable targeting of previously undruggable or heterogeneous disease sites [30,164,165,166].

The development of multifunctional and theranostic PINPs, which combine therapeutic delivery, real-time imaging, and diagnostic capabilities in a single platform, is expected to accelerate. For instance, gold and iron oxide nanoparticle cores can simultaneously serve as drug carriers and imaging agents (CT, MRI), while peptide coatings enable tumor targeting or stimuli-responsive release. Such systems allow for real-time drug distribution and treatment response monitoring, offering a personalized and adaptive therapeutic strategy [49,50,115,116,130].

AI and ML technologies are poised to revolutionize the design and optimization of PINPs by predicting structure–activity relationships, toxicity, biodistribution, and target affinity. Machine learning models trained on large datasets of peptide sequences and nanoparticle formulations can accelerate the discovery of high-performance candidates by simulating their interactions with biological targets or predicting in vivo behaviors [167,168,169,170]. This approach reduces trial-and-error experimentation, lowers cost, and shortens development timelines. In addition, ML and AI could assist researchers in optimizing the formation of PINPs by predicting biomineralization efficiency based on peptide sequences and the resulting AuNP optical characteristics [171].

Finally, advancing PINPs toward clinical translation requires addressing scalability, regulatory hurdles, and long-term safety. Future research must focus on scalable green synthesis methods, GMP-compliant production, and rigorous preclinical models that mimic human physiology. Integration of biocompatible materials, robust in vivo tracking, and clear regulatory guidelines will be crucial. Moreover, incorporating PINPs into combination therapies (e.g., chemo–immunotherapy) and personalized medicine frameworks could unlock their full clinical potential.

In conclusion, interdisciplinary innovation will shape the next generation of PINPs by combining molecular design, nanotechnology, data science, and translational medicine. By bridging molecular design principles (as outlined in Section 12) with translational science, peptide–MNPs stand poised to redefine the next generation of nanotherapeutics and move from experimental platforms into clinically viable tools for precision healthcare.

## 14. Conclusions

PINPs represent a rapidly evolving frontier in nanomedicine, offering an elegant fusion of biological specificity and physicochemical functionality. This review has outlined the significant advances and current trends in PINP research, including their use as targeted drug delivery vehicles, self-assembling platforms, capping and reducing agents, and stimuli-responsive systems. The integration of peptides, which bring inherent biocompatibility, receptor specificity, and responsiveness, with metal cores such as gold, silver, iron oxide, selenium, and gadolinium has enabled the creation of multifunctional nanostructures capable of precise, efficient, and traceable therapeutic delivery.

Among the key findings, peptide-mediated synthesis techniques offer eco-friendly and scalable routes for nanoparticle formation. At the same time, advanced characterization tools like TEM, FTIR, and zeta potential analysis continue to provide insights into PINP architecture and behavior. Functionalization with targeting peptides enhances site-specific accumulation, while stimuli-responsive designs allow on-demand drug release, further improving therapeutic outcomes and reducing systemic side effects. Importantly, these systems’ biocompatibility and partial biodegradability make them attractive candidates for clinical development.

Despite their promise, challenges remain, including stability in biological fluids, immunogenicity, toxicity at higher doses, and reproducibility during large-scale production. Furthermore, regulatory uncertainties and a lack of standardized protocols for evaluation have hindered clinical translation. Addressing these barriers will require concerted efforts from chemists, biologists, engineers, data scientists, and clinicians.

Looking forward, the future of PINPs lies in advanced peptide engineering, theranostic integration, and AI-driven design, which collectively hold the potential to deliver intelligent, patient-tailored nanomedicines. As research becomes increasingly interdisciplinary, collaborations across diverse scientific domains will be critical in transforming PINPs from experimental platforms into clinically viable tools for precision drug delivery and personalized therapy. By implementing rational design rules that connect peptide structure with intracellular delivery function, and by bridging such molecular design with translational science, PINP systems can overcome current barriers and achieve maximal efficacy in vivo. PINPs thus stand poised to redefine the next generation of nanotherapeutics, translating the promise of nanomedicine into tangible clinical outcomes.

## Figures and Tables

**Table 1:** Summary of common MNPs used in biomedical applications.

MNP Type	Unique Properties	Biomedical Applications [Ref]
Gold (AuNPs)	High stability and biocompatibility; strong SPR (~520 nm)	Photothermal therapy, imaging; easily functionalized for targeted drug delivery [14].
Silver (AgNPs)	Potent antimicrobial and anticancer activity (ROS generation)	Wound healing, antibacterial coatings; co-delivery with antibiotics or anticancer drugs for synergistic effect [15].
Iron Oxide (Fe_3_O_4_)	Superparamagnetic; MRI contrast enhancement	Magnetic targeting of drugs; theranostics combining MRI imaging and drug delivery [16].
Zinc Oxide (ZnO)	Dissolves in acidic environments; generates ROS in tumors	Tumor-specific drug release and cytotoxicity due to pH sensitivity [17].
Selenium (SeNPs)	Antioxidant and immunomodulatory; induces apoptosis in cancer cells	Delivery of chemotherapeutics; treatment of cancer, cardiovascular, and inflammatory diseases [18].
Gadolinium (GdNPs)	Paramagnetic, excellent MRI contrast; relatively heavy metal	Theranostics: combined MRI and drug delivery or photothermal therapy [19].

**Table 2:** Structural peptide classes and their roles in PINP systems.

Peptide Class	Structural Features	Roles in PINPs [Ref]
Linear	Unbranched amino acid chain	Easily modified; act as capping agents or templates (e.g., RGD-peptide for targeting); modulate NP size and functionality [35].
Cyclic	Head-to-tail or side-chain (disulfide/lactam) cyclization	Rigid and protease-resistant; provide high-affinity binding; can reduce and stabilize NPs simultaneously (e.g., [WR] peptides for AuNPs) [26,35].
Amphipathic (CPPs)	Spatially segregated hydrophobic/hydrophilic residues (often α-helical)	Facilitate membrane penetration and endosomal escape; stabilize MNPs in aqueous media; common cell-penetrating motifs (HIV-TAT, penetration) enhance uptake of MNP cargos [36].
Self-assembling	Sequences forming β-sheet or fibrillar structures (often aromatic)	Form nanofibers or hydrogels that template inorganic growth; encapsulate drugs; enable stimuli-responsive release (e.g., fibrils with cysteines nucleate metal nanoparticles) [37].

**Table 3:** Influence of peptide morphology on PINP intracellular delivery stages.

Peptide Morphology	Nanoparticle Uptake Mechanism	Endosomal Escape Ability	Advantages	Limitations
Linear peptide coating	Primarily receptor-independent or nonspecific endocytosis	Low, often leads to entrapment in endo-lysosomal compartments [38]	Simple design; easy synthesis and functionalization	Poor endosomal escape; cargo degradation in lysosomes
Cyclic peptide coating	Targeted receptor-mediated endocytosis due to high binding affinity [24]	Low, generally remains in vesicles without membrane-lytic properties [39]	High structural rigidity; protease resistance; selective targeting	Limited membrane-disruptive capacity [39]
Amphipathic peptide coating	Endocytosis (often receptor-mediated if targeting domain is present) or direct translocation bypassing endocytosis [40]	High, disrupts endosomal membranes for cytosolic release	Strong membrane interaction; efficient endosomal escape; enables delivery to cytosol/nucleus	May lack targeting specificity; risk of nonspecific membrane disruption [39]

**Table 4:** Functional classification of peptides in PINPs.

Function Category	Description and Examples [Ref]
Targeting Ligands	Peptides that bind overexpressed receptors (e.g., RGD for integrins, CRGDK for NRP-1) direct PINPs to tumors, e.g., CRGDK–AuNPs show enhanced tumor accumulation; dual-targeted AuNPs carrying both a homing peptide and drug peptide improve delivery efficiency [43].
Capping/Stabilizing Agents	Peptides that bind metal surfaces (thiol, amine, carboxyl) to prevent aggregation and add biofunctionality. For example, cysteine-containing peptides cap AuNPs via Au–S bonds; peptides embedding RGD sequences provide stability and integrin targeting [51].
Reducing Agents	Redox-active peptides reduce metal ions to NPs under mild conditions. For instance, peptides with multiple Trp reduce Au^3+^ while binding the NP, yielding stable AuNPs (e.g., [WR] peptides) [26].
Therapeutic/Functional	Peptides that carry bioactivity or responsive elements (e.g., AMPs, enzyme-cleavable linkers) to add functionality (overlapping) [52,53,54].

**Table 5:** Comparison of MNP synthesis approaches and reducing/capping agents.

Approach	Reducing/Capping Agent	Notes/Examples [Refs]
Chemical (Turkevich)	Sodium citrate (mild reductant + weak capping)	Classic AuNP synthesis (10–20 nm); citrate reduces Au^3+^ to Au^0^. Produces stable colloids, but organic solvents or byproducts are possible [89].
Chemical (borohydride)	NaBH_4_ (strong reductant); surfactant capping	Produces very small Ag/Au NPs (<5 nm); fast reduction but often leaves toxic by-products [90].
Plant extracts (bio)	Polyphenols (e.g., flavonoids)	Green synthesis: leaf/fruit extracts reduce Au/Ag ions to NPs. E.g., *Camellia* extract yields AuNPs. Eco-friendly and scalable, product variability depends on extract composition [91,92].
Peptide-mediated (green)	Functional peptides (Tyr, Trp, Cys rich)	Peptides reduce and cap in one step, e.g., cyclic [WR] peptides form AuNPs with integrated targeting. Mild conditions yield biocompatible, functionalized NPs [26].
Physical (laser ablation)	None (requires energy input)	Generates pure MNPs in solution without chemical reagents. Can control size by laser parameters; high energy cost, specialized equipment [93].

**Table 6:** Comparative summary of peptide classes in intracellular delivery PINP systems.

Peptide Class	Typical Cargo and Role	Delivery Efficiency (Examples)	Key Limitations
Cell-Penetrating Peptides (CPPs), e.g., TAT, penetratin, poly-Arg (R_9_)	Broad range: small drugs, siRNA, proteins, often used to ferry cargo across cell membranes (non-specific uptake enhancer).	High cellular uptake: For instance, TAT-modified AuNP–drug conjugates significantly delayed tumor growth in vivo compared to free drug [71].	Non-specific targeting: CPPs enter many cell types, potentially causing off-target effects. Often become sequestered in endosomes (if not combined with endosomolytic element). Highly cationic CPPs can be cytotoxic at high density (membrane disruption, hemolysis) [153].
Targeting peptides (homing ligands), e.g., cRGD, iRGD, LyP-1, F3 peptide	Typically, small molecules or drugs attached to NPs; peptides guide NPs to specific cell-surface receptors (tumor vasculature, cancer cells, etc.). Often used for targeted drug delivery.	Enhanced tissue/cell specificity: Peptide-guided NPs achieve high accumulation at target sites, e.g., doxorubicin-loaded AuNPs functionalized with cRGD showed greater tumor uptake and lower off-target toxicity than free drug [153]. RGD-decorated NPs increased tumor localization in integrin-rich tumors (vs. non-targeted) [154]. Some targeting peptides (e.g., iRGD) also promote deep tumor penetration and enhance chemotherapeutic efficacy in vivo [154,155].	Dependency on receptor expression: Only effective if target receptors are highly expressed; variability in patients’ tumors can reduce efficacy. Uptake is typically via receptor-mediated endocytosis, and cargo can remain entrapped without an additional escape mechanism. Possible immunogenicity for larger targeting peptides or if the human sequence is foreign.
Amphipathic and fusogenic peptides, e.g., INF7 (derived from HA2), GALA, KALA, LK15, histidine-rich peptides	Often used alongside CPPs or targeting ligands to promote endosomal escape of nucleic acids (siRNA/DNA), toxins, or other therapeutics. Some also serve as primary CPPs (transportan, arginine-rich AMPs).	Greatly improved cytosolic delivery: For example, histidine-rich CPPs enhance endosomal escape via proton sponge swelling and membrane disruption, significantly boosting functional delivery efficiency for proteins and nucleic acids [156]. Conjugation of histidine-rich peptides to nanoparticles improved cytosolic access, confirmed by leakage assays and microscopy, without significant toxicity. Fusogenic peptide strategies prevent lysosomal degradation and can increase protein cytosolic delivery several-fold [157].	Potential cell toxicity: If too potent, they can disrupt plasma membranes or cause global membrane damage. Often pH-dependent; efficacy may vary with endosomal maturation conditions. Usually not cell-specific (need to be targeted to the endosome of specific cells via another ligand). Peptide sequences may risk complement activation or immunogenicity if administered systemically in high doses.
Stabilizing/capping peptides, e.g., cysteine-rich peptides, glutathione, di- or tripeptides (CG, YY)	Serve as surface coatings to stabilize metal NPs (prevent aggregation) and can also reduce metal ions during “green” synthesis. Cargo is typically the NP itself (which then carries drugs or imaging agents); sometimes a drug is co-conjugated.	Improved colloidal stability and circulation: Cysteine-capped AuNPs suppress protein aggregation under heat stress and maintain nanoparticle stability [158]. Glutathione self-assembles into dense, ordered shells on metal nanoparticles, preventing aggregation and enhancing biocompatibility [159]. PEG-cofunctionalized gold nanoparticles with glutathione exhibit prolonged circulation times and reduced opsonization in vivo [160].	May reduce cellular uptake: Highly hydrophilic or “stealth” capping peptides (or PEGylated peptides) can inhibit interaction with target cells (lower uptake). Often no active targeting or uptake function—they may need additional targeting or CPP ligands for efficient delivery. Peptide capping layers must remain intact until reaching target site (risk of premature desorption in blood).
Self-assembling peptide systems, e.g., peptide nanofibers, peptide amphiphiles that nucleate metals [161,162]	Peptides form the nanoparticle scaffold or template; they can carry drugs within peptide assemblies or nucleate metal NP growth in situ. Cargo can be drugs co-assembled, or the peptide–metal hybrid itself acts as the therapeutic	One-step construction and high payload: Peptide-driven assembly can create uniform nanostructures with high peptide density, simplifying functionalization. For example, short dipeptides were used to simultaneously reduce gold ions and cap the resulting AuNPs, achieving a high peptide payload per particle [161]. Some cyclic peptide nanospheres can encapsulate drugs and release them in response to stimuli [162].	Polydispersity and reproducibility issues: Self-assembly is sensitive to conditions; slight changes can alter NP size/shape, making consistency a challenge [163]. These systems may lack in vivo stability, and peptide assemblies might disassemble or be enzymatically degraded before reaching the target. Tuning their biodegradation rate is complex. Also, without further modification, purely peptide-based NPs might have limited target specificity or might trigger immune responses if the assembled structures are recognized as foreign [162].

## Abbreviations

The following abbreviations are used in this manuscript:

PINPs	Peptide-based inorganic nanoparticles
AuNPs	Gold nanoparticles
SeNPs	Selenium nanoparticles
GdNPs	Gadolinium nanoparticles
